# Platelet activity and hypercoagulation in type 2 diabetes

**DOI:** 10.1186/s12933-018-0783-z

**Published:** 2018-11-02

**Authors:** Lesha Pretorius, Greig J. A. Thomson, Rozanne C. M. Adams, Theo A. Nell, Willem A. Laubscher, Etheresia Pretorius

**Affiliations:** 1 Department of Physiological Sciences, Stellenbosch University, Stellenbosch Private Bag X1, Stellenbosch, 7602 South Africa; 2 Central Analytical Facilities, Fluorescence Imaging Unit Stellenbosch University, Stellenbosch Private Bag X1, Stellenbosch, 7602 South Africa; 3 Department of Electronic and Electric Engineering, Faculty of Engineering, Stellenbosch University, Stellenbosch Private Bag X1, Stellenbosch, 7602 South Africa

**Keywords:** Type 2 diabetes, Platelets, GPIIb/IIIa receptor, Microparticles

## Abstract

**Background:**

A strong correlation exists between type 2 diabetes mellitus (T2DM) and cardiovascular disease (CVD), with CVD and the presence of atherosclerosis being the prevailing cause of morbidity and mortality in diabetic populations. T2DM is accompanied by various coagulopathies, including anomalous clot formation or amyloid fibrin(ogen), the presence of dysregulated inflammatory molecules. Platelets are intimately involved in thrombus formation and particularly vulnerable to inflammatory cytokines.

**Methods:**

The aim of this current study was therefore to assess whole blood (hyper)coagulability, platelet ultrastructure and receptor expression, as well as the levels of IL-1β, IL-6, IL-8 and sP-selectin in healthy and diabetic individuals. Platelet morphology was assessed through scanning electron microscopy (SEM), while assessment of GPIIb/IIIa receptor expression was performed with confocal microscopy and flow cytometry with the addition of FITC-PAC-1 and CD41-PE antibodies. IL-1β, IL-6 and IL-8 and sP-selectin levels were assessed using a multiplex assay.

**Results:**

In T2DM there is significant upregulation of circulating inflammatory markers, hypercoagulation and platelet activation, with increased GPIIb/IIIa receptor expression, as seen with flow cytometry and confocal microscopy. Analyses showed that these receptors were additionally shed onto microparticles, which was confirmed with SEM.

**Conclusions:**

Cumulatively, this provides mechanistic evidence that pathological states of platelets together with amyloid fibrin(ogen) in T2DM, might underpin an increased risk for cardiovascular events.

## Introduction

Type 2 diabetes mellitus (T2DM) has become one of the most prevalent and costly chronic diseases of lifestyle [1, 2]. Statistics from the World Health Organisation (Nov, 2017) indicated an increased incidence of diabetes from 108 million (1980) to 422 million (2014). The highest incidence mostly occurs in regions dominated by developing countries due to westernization and urbanization [2]. According to the International Diabetes Federation (IDF) these statistics are expected to further increase to 642 million diagnosed individuals between the ages of 20–79 years in 2040 [2], more than 6% of the entire population.

Evidence demonstrates a strong correlation between T2DM and cardiovascular disease (CVD), with CVD and the presence of atherosclerosis being the prevailing cause of morbidity and mortality in diabetic populations [1, 3]. Furthermore, The Insulin Resistance Atherosclerosis Study (2002) confirmed the association of chronic inflammation with development of T2DM, as well as the relationship between the resultant insulin resistance and progression of atherosclerosis [4]. It is well known that a dysregulated low-grade systemic inflammatory *milieu* is present in T2DM, including C-reactive protein (CRP), tissue factor, interleukins (IL-1β, IL-6 and IL-8) and tumour necrosis factor alpha (TNF-α) [5–8]. These elevated circulating inflammatory markers are associated with dyslipidaemia and atherosclerosis (albeit markers of many other inflammatory diseases [9]), and are thought to be potential predictors of the development of T2DM [4, 10, 11].

Previously our group has shown that many chronic, inflammatory diseases, including T2DM, are accompanied by various coagulopathies, which manifest as anomalous clot formation in the form of ‘dense matted deposits’ that might arise in circulation due to the presence of dysregulated inflammatory markers [7, 12–14]. More recently we have shown that in T2DM, these clots are amyloid in nature, where the actual fibrin molecules have undergone structural alterations. This was demonstrated using fluorescent amyloid protein markers which were added to platelet-poor plasma (PPP) from individuals with T2DM [15, 16]. Considering the cytotoxic characteristics of amyloids and many of the sequelae of chronic T2DM involving damage to cells, the focus of the current paper is to study platelet activation in the presence of aberrant fibrin(ogen) in diabetic individuals.

The platelet membrane consists of glycoproteins, integrins, phospholipids and other receptors [17]. Major platelet receptors include G-protein coupled receptors, tyrosine kinase adhesive receptors, integrins, leucine-rich adhesion receptors and immunoglobulin superfamily adhesion receptors [17].

Upon activation, platelets undergo conformational changes that result in cytoplasmic foot-like extensions known as pseudopodia, also known as simple contact-level activation [18]. However, further activation, degranulation and platelet adhesion is required during primary haemostasis [19]. The platelet membrane flattens in a “fried-egg-like” silhouette, in order to cover an increased surface area. Activated platelets also provide a negatively charged pro-coagulant surface, to facilitate aggregation [20].

The formation of circulating platelet-derived microparticles might be of interest in T2DM. These microparticles are microvesicles, approximately 0.02–0.1 μm in diameter [21], that are released by platelets upon activation [22]. They have been shown to possess most of the membrane proteins and receptors found on platelets including P-selectin, GPIb/CD41 [23] and GPIIb/IIIa. Formation of microparticles is associated with the loss of asymmetry of the platelet phospholipid membrane i.e. externalization of phosphatidylserine [24, 25]. Platelet-derived microparticles promote platelet interaction with the sub-endothelial matrix [26] and are thought to be involved in thrombin generation [27]. Elevated levels of these microparticles are observed in various pathological conditions such as myocardial infarctions [25].

Activation of platelets also induces the rapid translocation and expression of P-selectin, which is stored within the platelet α-granules, to the cell surface [28, 29]. P-selectin plays a key role in haemostasis as it mediates the adhesion of activated platelets to neutrophils and monocytes to facilitate the innate immune response, as well as inducing platelet-to-platelet binding and aggregation [30]. Thus, P-selectin proteins can be secreted into circulation, now called soluble P-selectin (sP-selectin), as apart of platelet-derived microparticles or as free spliced versions of the protein. Consequently, an increase in sP-selectin occurs upon platelet activation [31], and can therefore possibly be used as a surrogate marker of platelet activation.

The aim of this current study was to assess whole blood (WB) (hyper)coagulability, platelet ultrastructure, as well as the levels of three interleukins (IL-1β, IL-6 and IL-8) and sP-selectin in healthy and diabetic individuals. Platelet morphology was assessed through scanning electron microscopy (SEM) of platelet rich plasma to show platelet ultrastructure and interactions. IL-1β, IL-6 and IL-8 and sP-selectin levels were assessed with a multiplex assay. We also assessed GPIIb/IIIa receptor expression with confocal microscopy and flow cytometry with the addition of FITC-labelled monoclonal antibodies-PAC-1 [32–34], correlated to CD41 expression on platelets.

## Materials and methods

### Ethics, consent and permissions

Ethical clearance was obtained from the Health Research Ethics Committee (HREC) of Stellenbosch University (Ethics Reference: 6329). Volunteers provided written informed consent for sample use and data publication, after which whole blood samples were collected in citrated tubes.

### Participants

A total of 60 healthy age-matched volunteers (refer to Table 1 for sample demographics) were recruited with the following inclusion criteria: (i) non-smokers (ii) no history of thrombotic disorders, and (iii) were not on any chronic antiplatelet therapy/anticoagulant medication or any contraceptive/hormone replacement therapy. Similarly, whole blood samples were collected from 51 individuals diagnosed with type 2 diabetes mellitus and cardiovascular disease. Diabetic volunteers were recruited and blood samples were obtained as part of standard care during their routine visit to their medical practitioner, at the MediClinic Hospital, Stellenbosch. The inclusion criteria for this group included: (i) a confirmed diagnosis of type 2 diabetes with cardiovascular disease, and (ii) males and females older than 35 years. To limit and exclude confounding factors, volunteers from both healthy and diabetic groups were only included if they did not have tuberculosis, HIV or any malignancies. Additionally, diabetic volunteers on GPIIb/IIIa inhibitors were excluded from the study.

**Table 1:** Demographics of healthy (n = 60) and type 2 diabetic (n = 53) volunteers

	Healthy individuals (n = 60)		Diabetic individuals (n = 53)		*p*-values
Gender	Male (n = 22), Female (n = 38)		Male (n = 27), Female (n = 26)		
Age (years)	59 ± 1.64 (n = 60)		64 ± 1.8 (n = 52)		
HbA1c (%)	5.2 ± 0.07 (n = 58)		8.9 ± 0.34 (n = 51)		< 0.0001

Data expressed as mean ± SEM. No significant correlation was observed between age and HbA1c between the healthy and diabetic samples (Pearson-test)

Medications were recorded in conjugation with biomedical parameters, with the most prevalent amongst diabetes patients including Metformin^®^ (n = 40) oral hypoglycaemic, simvastatin (n = 21) for cholesterol regulation, and Coversyl^®^ (n = 13) for blood pressure regulation

### Sample preparation

Whole blood was kept at room temperature in citrate tubes for thromboelastographic (TEG) analyses. To create platelet-rich plasma (PRP) for electron microscopy, confocal and flow cytometry, citrated blood samples were centrifuged at 150×*g* for 10 min at room temperature (± 22 °C) to separate PRP from other blood constituents. To create PPP for multiplex analysis, citrated whole blood samples were centrifuged at 3000×*g* for 15 min at room temperature to separate PPP from other blood constituents. The PPP was stored at – 80 °C, until day of multiplex analyses.

### Thromboelastography

TEG analysis was performed on naïve (untreated) whole blood samples. A TEG analysis requires the addition of 20 μL calcium chloride (CaCl_2_) and 340 μL of WB to a disposable TEG cup, which is according to manufacturer instructions and previously published papers [35, 36]. CaCl_2_ reverses the effect of the sodium citrate (citrated tube), which then initiates the coagulation cascade. Seven viscoelastic TEG parameters were used to assess coagulation efficiency in this study. Thromboelastographies were performed using the Thromboelastograph 5000 Hemostasis Analyzer System, configured and used according to the manufacturer’s protocol.

### Multiplex cytokine analysis

Platelet-poor plasma from control (n = 21) and T2DM (n = 24) volunteers were analysed in duplicate using the Invitrogen’s Inflammation 20-Plex Human ProcartaPlex™ Panel (catalogue number: EPX200-12185-901). Briefly, 25 µL of PPP and internal controls were incubated with magnetic beads prior to a series of wash steps. 25 μL of detection antibody was added and incubated for 30 min before 50 μL of Streptavidin-PE was added. The 96-well plate was then analysed using Bio-Plex^®^ 200 system (BioRad) with inflammatory markers being measured in pg mL^−1^.

### Scanning electron microscopy

10 μL of PRP is used to prepare a scanning electron microscopy smear. Sufficient time is allowed for PRP sample attachment to the 10 mm round glass slide before the addition of 10× Gibco™ PBS (phosphate-buffered saline), pH 7.4 (ThermoFisher Scientific, 11594516). All smears were fixed with 4% paraformaldehyde in PBS for at least 30 min, followed by three PBS washes before fixation with 1% osmium tetroxide (Sigma-Aldrich, 75632) in double distilled H_2_O for an additional 30 min. The samples were again washed three times with PBS. An ethanol series dehydration was performed in which samples were washed in 30%, 50%, 70%, 90% and 100% ethanol for 3 min each time. Sample dehydration is completed with 99.9% hexamethyldisilazane *ReagentPlus*^®^ (Sigma-Aldrich, 379212) treatment for 30 min, after which the samples are left to air dry in a fume hood overnight (± 16 h). Dried samples are mounted on glass microscope slides with double-sided carbon tape before the final carbon coating is applied. Scanning electron microscopy ultrastructural analysis of PRP samples was performed on the Zeiss MERLIN™ field emission scanning microscope located in the Central Analytical Facility (CAF) Electron Microbeam Unit, Stellenbosch University. Micrographs were captured using high resolution InLens capabilities at 1 kV.

### Flow cytometry

For platelet staining, 100 μL of PRP was aliquoted into 12 × 75 mm round bottom tubes (BD Biosciences, 352063). Thereafter, 20 μL of PAC-1 FITC (BD Biosciences, 340507) and 20 μL of CD41 PE (Beckman Coulter, IM1416U) stored in a phosphate buffered saline storage solution with gelatin and 0.1% sodium azide, were added to the PRP and gently mixed by pipetting. The samples were incubated in a dark environment for 30 min at room temperature. After incubation, 500 μL of PBS was added to each tube and the samples were analysed on the BD FACSAria IIu cell sorter located in the CAF Fluorescence Microscopy Unit, Stellenbosch University. For each sample, a minimum of 30,000 events were acquired and all signal were gated. The addition of prostaglandin (which is usually added to prevent platelet activation during a second step where a platelet pellet is needed), was omitted since PRP was obtained by centrifuging WB only once, at a very low relative centrifugal force (150×*g*). For compensation, single stained platelets were used to determine optimal voltages and Anti-Mouse Ig compensation beads (BD Biosciences, 552843) were used to determine the compensation matrix. To ensure the consistency and reproducibility of the data, application settings were set and applied to the experiment and eight peak beads were used as a secondary measure. Platelets were identified and gated using SSC vs CD41-PE dot plot and the PAC-1 positive and negative cells were identified from this population. All analyses were performed using FlowJo v10.4.1, and data were exported to Microsoft Excel for further analysis.

### Confocal microscopy

The platelet staining procedure for confocal microscopy is identical to that used for flow cytometric analysis. Further sample processing included the deposition of 6 μL of fluorescently stained PRP sample on a microscope slide 10 min prior to viewing, to allow platelets to settle. The Zeiss LSM 780 ELYRA PS1 confocal microscope with Super-Resolution Structured Illumination Microscopy (SR-SIM) was used in this study (CAF, Fluorescence Microscopy Unit, Stellenbosch University).

### Statistical analysis

All statistical analyses were performed using GraphPad/Prism v7. Data was checked and tested for normality using the Shapiro–Wilk normality test. All data is either expressed as means and standard deviations, or medians and interquartile ranges. To analyse differences in TEG parameters between diabetic and healthy individuals, an unpaired t-test was performed for parametric, and the Mann–Whitney test for non-parametric data between the two groups. Statistical significance was accepted at p < 0.05. For the biomarker analysis, the ROUT method for detecting outliers was used in cases where data was not normally distributed. A modified t-test (Welch correction) was performed on the cleaned data.

## Results

The results of the TEG analysis (Table 2) show significant differences between all parameters assessed. Compared to healthy individuals, diabetic individuals showed significantly slower reaction time (R-value), clot kinetics (*K*) and time to maximum rate of thrombus generation (TMRTG). Furthermore, diabetic individuals had significantly higher TEG values for the clot angle, maximum clot amplitude (MA), maximum rate of thrombus generation (MTRG) and total thrombus generation (TTG). This suggests a hypercoagulable state in T2D individuals.

**Table 2:** TEG results of the seven viscoelastic parameters assessing the efficiency of coagulation in naïve whole blood samples of healthy (n = 44) and diabetic (n = 26) volunteers

Parameter	Healthy individuals (n = 51)	Diabetic individuals (n = 36)	*p*-value
R-value	8.2 [7–9.8]	6.6 [4.7–8.4]	0.001
K	2.8 [2.2–3]	1.9 [1.6–2.5]	0.0004
A (angle)	59.7 [51.9–64]	68.8 [63.3–72.2]	< 0.0001
MA	58.8 [55.1–63.6]	66.1 [60.5–71.1]	0.0001
MRTG	4.6 [4.2–5.8]	7.4 [5.7–10.4]	< 0.0001
TMRTG	11.9 [9.8–13.6]	9.4 [7.9–12.1]	0.003
TTG	715.1 [616.9–877]	978.1 [774.8–1222]	0.0001

Data expressed as means and interquartile ranges. Parameters were compared using the non-parametric Mann–Whitney test

**Fig. 1:**
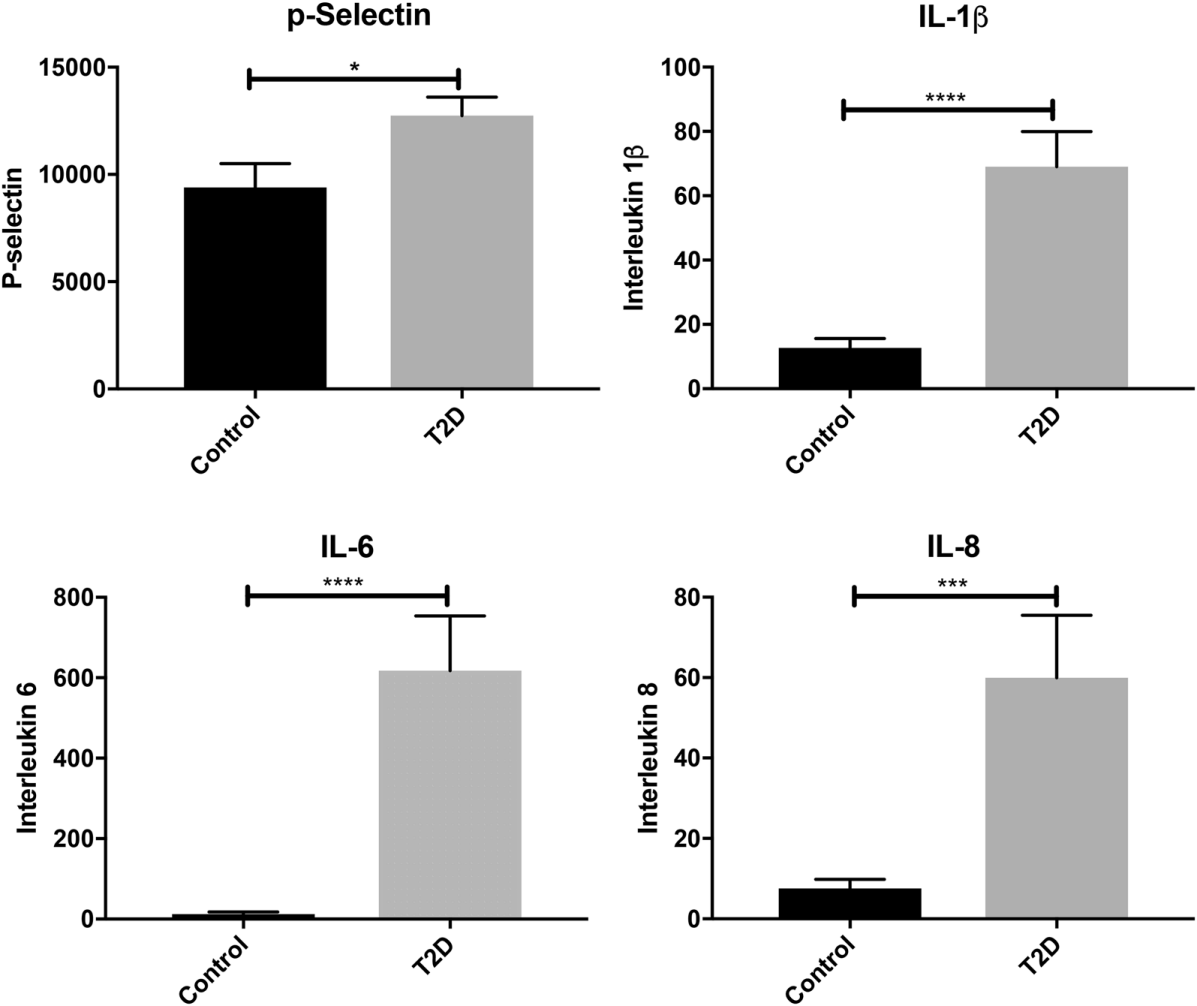
Graphs of circulating inflammatory markers: soluble P-selectin, IL-1β, IL-6, IL-8 (pg mL^−1^) in 25 µL of platelet-poor plasma of control (n = 21) and diabetic (n = 24) individuals using Invitrogen’s Inflammation 20-Plex Human ProcartaPlex Panel. Data expressed as mean ± SEM with *p < 0.05; ***p < 0.001 and ****p < 0.0001. Values in controls that were lower than detectable ranges were allocated ‘0’

Previously we have noted that when IL-1β, IL-6 and IL-8 is added to WB from healthy individuals, platelet hyperactivation is stimulated [37]. In the current analysis, we investigated the levels of these cytokines in our samples. Multiplex cytokine analyses confirmed the significant upregulation of circulating levels of IL-1β, IL-6 and IL-8 in PPP from T2DM individuals when compared to controls (Fig. 1). Moreover, sP-selectin, a plausible marker of platelet activation, was also significantly upregulated (p < 0.05) in the diabetic groups when compared to healthy individuals.

**Fig. 2:**
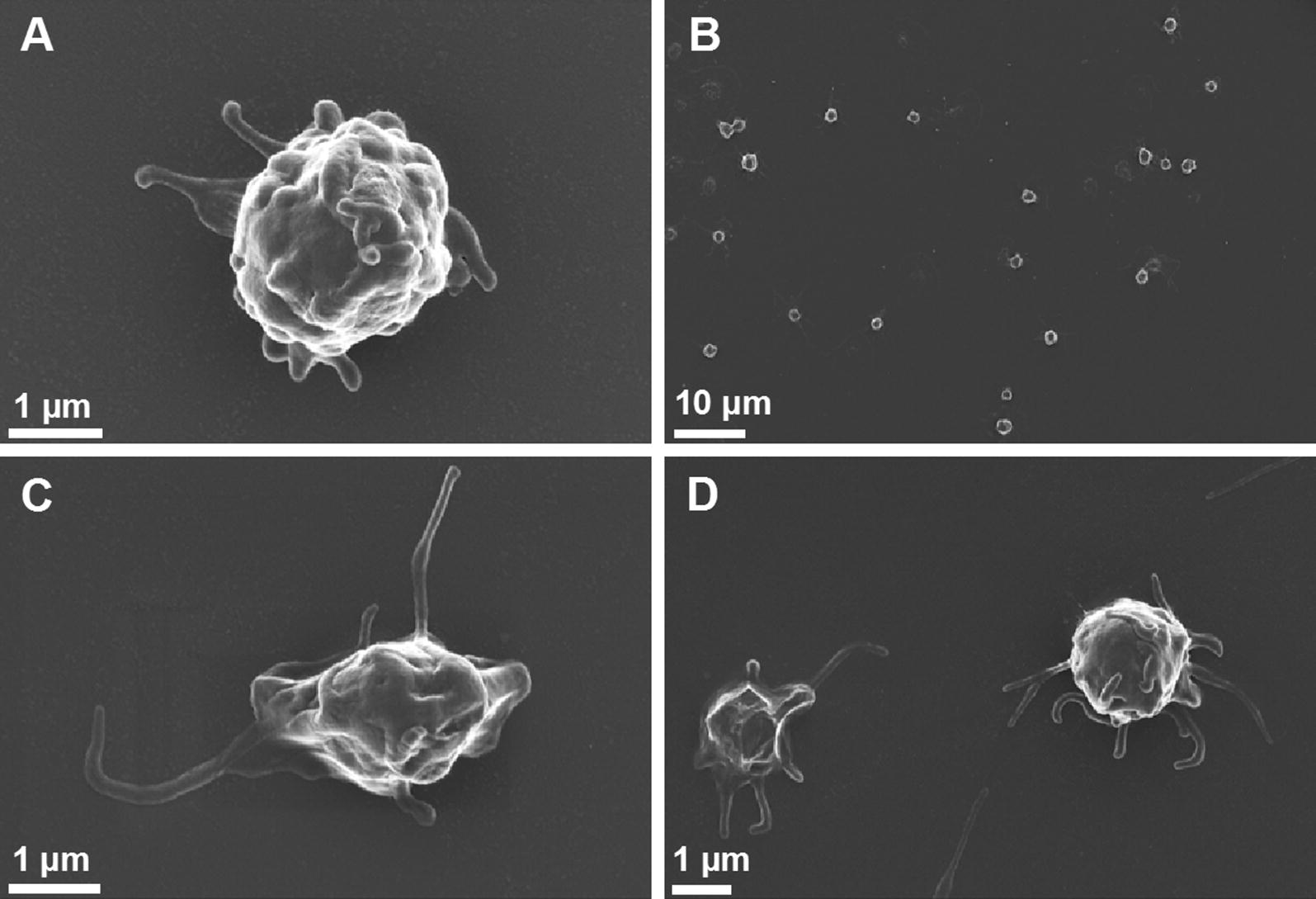
Scanning electron micrographs of platelets from healthy volunteers prepared from platelet-rich plasma depict rounded, slightly activated platelets with pseudopodia formations. **A**, **C** and **D** high magnification and **B** low magnification

**Fig. 3:**
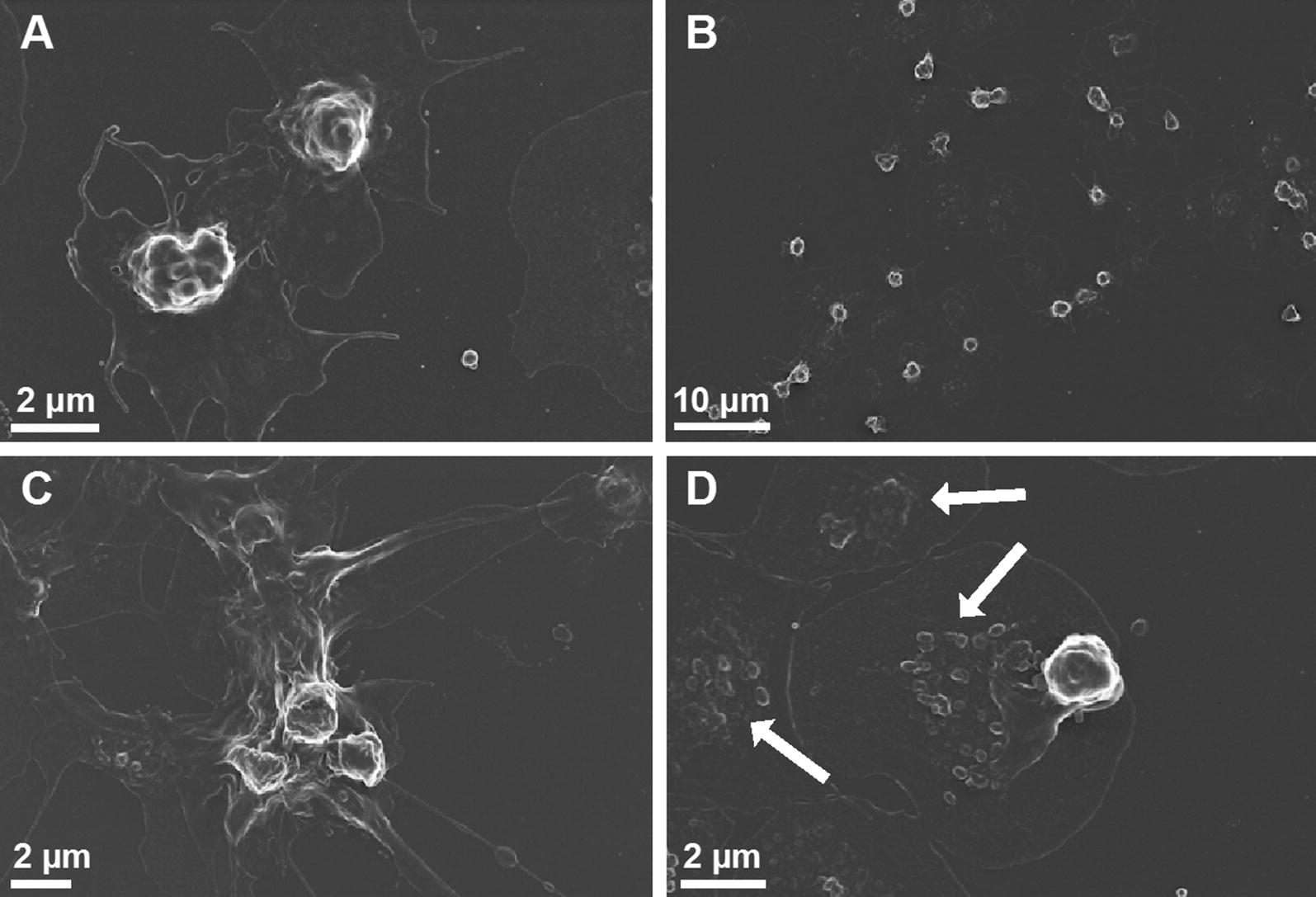
Scanning electron micrographs of platelets from diabetic volunteers prepared from platelet-rich plasma depicting platelet hyperactivation, membrane spreading, platelet-derived microparticle formation (see white arrows) and agglutination. **A**, **C** and **D** high magnification and **B** low magnification

Scanning electron microscopy analyses of platelets from control and T2DM volunteers are illustrated in Figs. 2 and 3. Morphologically, platelets from healthy individuals typically appear round, with slight pseudopodia formation, which is due to contact activation during the placement of the PRP onto the glass slide (Fig. 2). Contact activation could be a confounder, however, as the controls show limited activation on the cover slips, we believe that this serves as an appropriate baseline for excessive activation observed in T2DM. In the presence of inflammation, platelet hyperactivation, spreading and clumping may occur. This was also seen in samples from T2DM individuals, together with increased microparticle formation (Fig. 3).

**Fig. 4:**
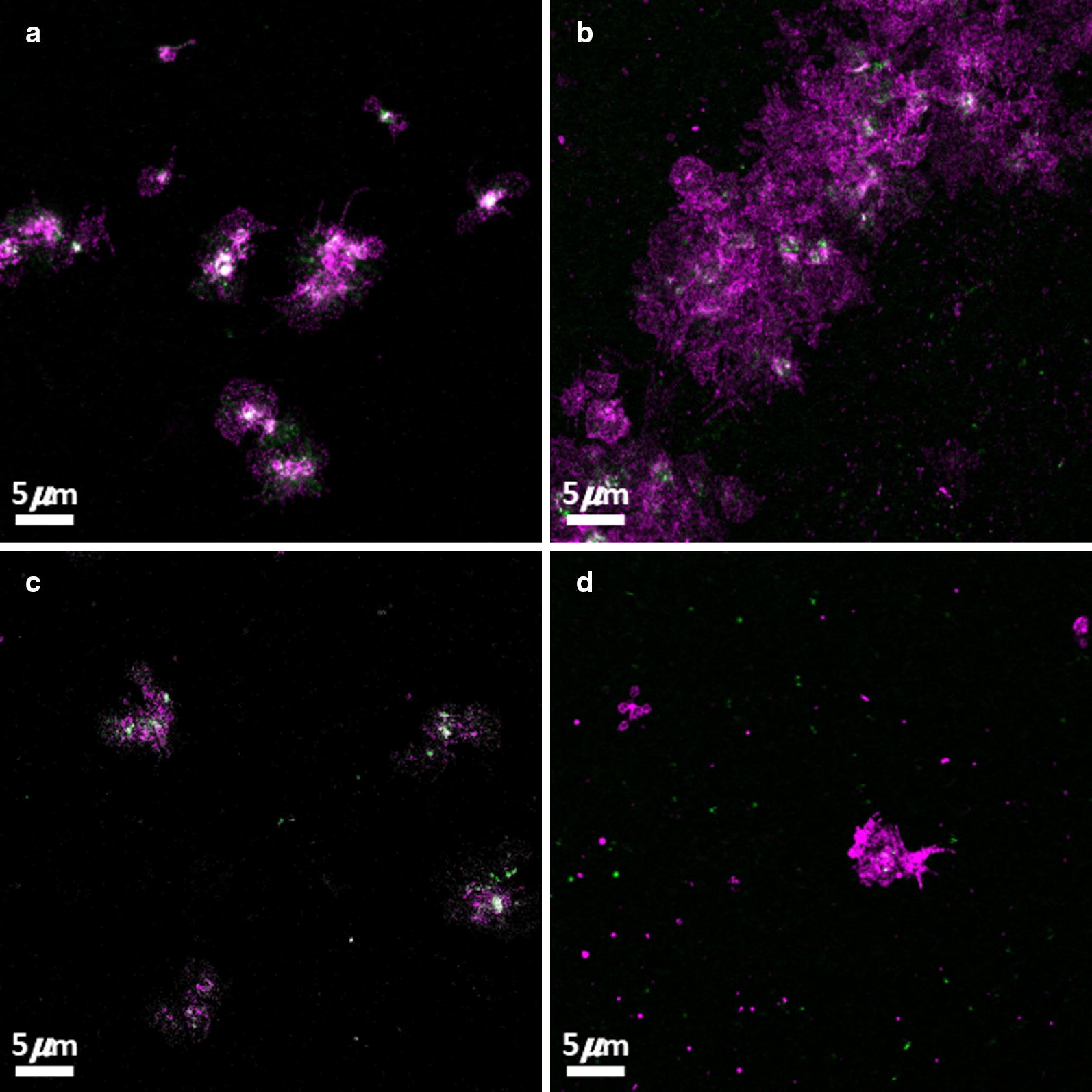
Confocal microscopy where platelets where incubated with CD41 (magenta) and PAC-1 (green). **a**, **c** Representative micrograph of platelets from healthy individuals with HbA1c values of 5.0% and 5.2% respectively. Both individuals also reported with CRP levels of < 1.00, indicative of no inflammation. **b**, **d** Representative micrographs of platelets from individuals diagnosed with type 2 diabetes mellitus. These individuals had HbA1c levels of 7.0% and 7.2% respectively

We also investigated the presence of the GPIIb/IIIa platelet receptor on platelets from both healthy and T2DM individuals. Confocal microscopy shows that in T2DM GPIIb/IIIa receptors (green signal) were present on both the actual platelets, but to a greater extent on the small platelet-derived microparticles (see Fig. 4d), while minimal if any shedding is seen in control samples. Additionally, diabetic samples displayed significant masses of platelet aggregates, indicative of the pro-thrombotic state of these individuals (see Fig. 4b). In micrographs of control PRP, signal overlap (white signal) is noted, indicating the presence of activated GPIIb/IIIa receptors on the actual platelets, while this is largely absent in the micrograph’s of PRP from T2DM individuals.

**Fig. 5:**
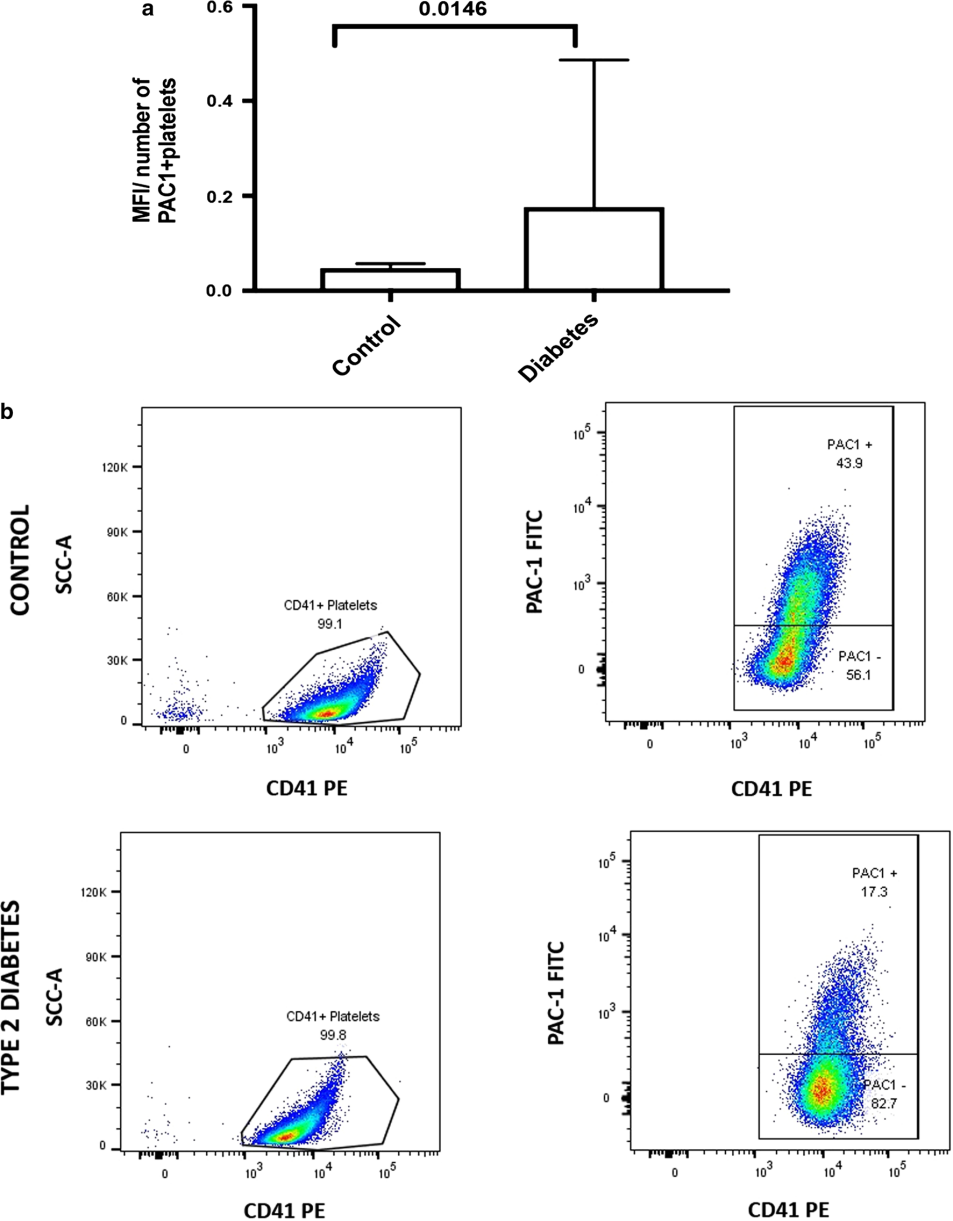
**a** Comparing the median fluorescent intensity per number of platelets positive for PAC-1 in healthy (n = 15) and diabetic (n = 20) samples using flow cytometry. This is representative of GPIIb/IIIa receptor expression. Data is expressed as medians and IQR; *significance (*p *= 0.0225). **b** Identification of PAC-1-positive platelets; platelets were gated for CD41. Note example of a control sample expressed 43.9% PAC-1 positive signal while the sample from a diabetic individual shows only 17.3% PAC-1 positive signal

The analyses by flow cytometry involved adding CD41-PE, as well as PAC-1 to PRP, and recording at least 30,000 events. We gated the singlet platelets by using FCS-A, and determined the number of platelets that showed PAC-1 signal. The number of platelets positive for PAC-1, as well as the median fluorescent intensity (MFI) for each sample was recorded. From these two values, we determined a coefficient of variation (CV) by dividing MFI by number of platelets with PAC-1 signal. Our results showed that in T2DM platelets, the CV is significantly more than in the control sample. This supports both our confocal and our SEM data. Furthermore, these results also support our significantly upregulated biomarker data, suggesting that the circulating upregulated cytokines in particular, result in a pro-inflammatory platelet environment, contributing to increased platelet receptor activity in T2DM. Flow cytometry results are shown in Fig. 5. Due to the small size of the platelet-derived microparticles, we could not quantify these using our flow cytometry system. Microparticles are also known to be pro-inflammatory and may additionally contain shed and activated receptors, as observed with confocal microscopy.

**Fig. 6:**
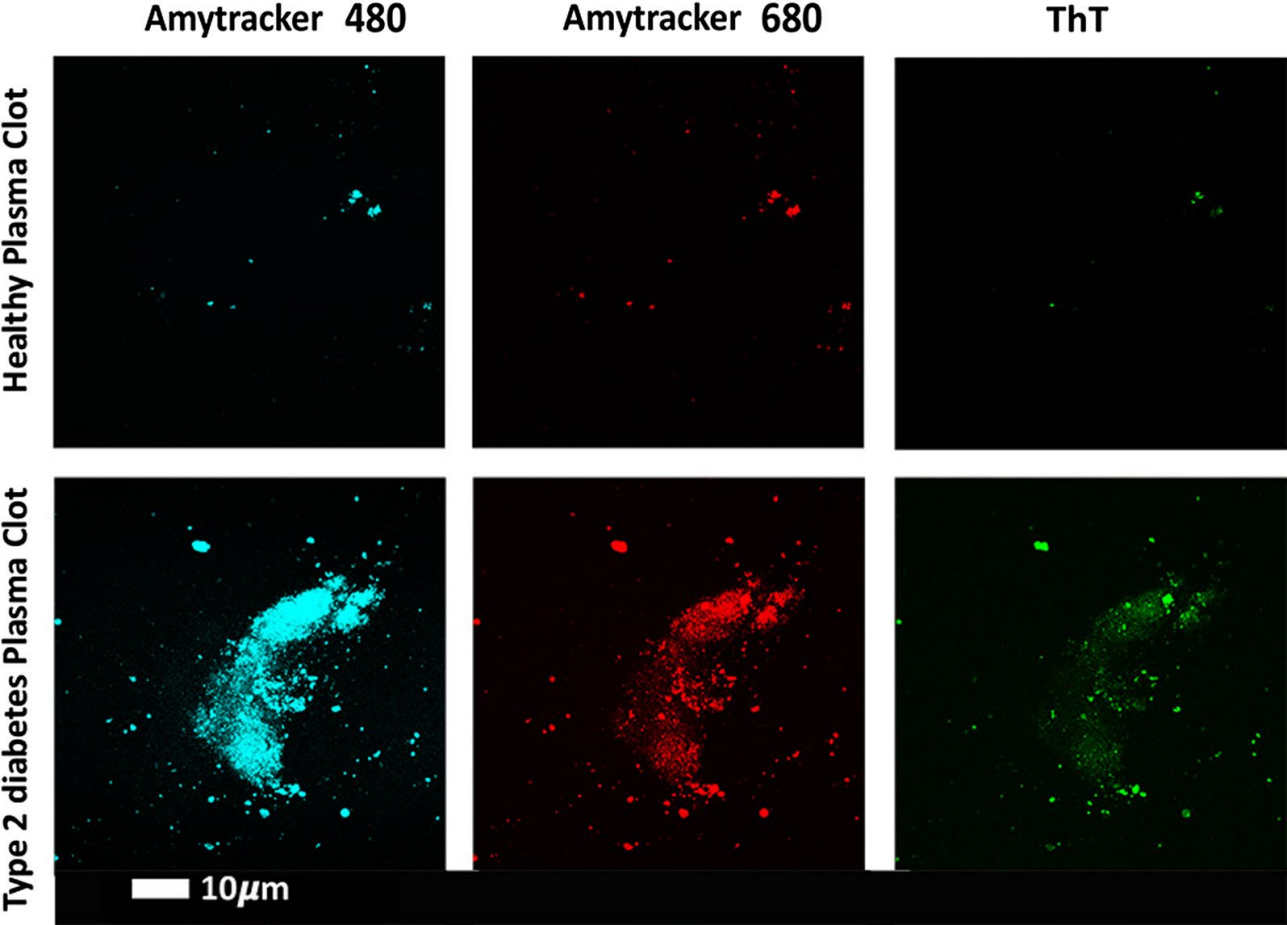
Fluorescent signals from platelet-poor plasma clots from a representative healthy and type 2 diabetic individual. Amyloid signal was detected with a Zeiss LSM 780 with ELYRA PS1 confocal microscope, using three fluorescent amyloid markers (Raw data from previously published papers [16, 38])

Previously we also showed that fibrin(ogen) in T2DM has an amyloid structure which contributes to the hypercoagulable nature of PPP in T2DM (see Fig. 6—unpublished raw data from [16, 38]). We have also previously shown that in T2D, the erythrocytes are more prone to be eryptotic (programmed cell death specific to erythrocytes) [37, 39]. The current results further confirm the presence of upregulated pro-inflammatory biomarkers, the presence of activated platelets, and increased presence of platelet receptors, resulting in a chronic systemic inflammatory profile that can be detected by analysis of both cellular and circulating biomarkers.

## Discussion

Analysis of the seven viscoelastic parameters with WB thromboelastography proved that significant differences in coagulation parameters exist between diabetic and healthy individuals (Table 2). A decreased clot reaction time (R-value) indicates accelerated clot initiation suggesting thrombus formation is more rapid in diabetic individuals. Decreased clot kinetics (*K*) would result in upregulated clot amplification; hence the forming clot will reach the specified strength (20 mm) quicker than a healthy individual. A decreased time to maximum rate of thrombus generation leads to a shorter time interval between clot initiation and maximum clot formation. Furthermore, an increased angle is generated from an increased thrombin burst, which results in upregulated fibrin cross-linking. Similarly, an increased maximum clot amplitude indicates that diabetic individuals display increased platelet and/or fibrinogen interaction, thus a denser, more rigid clot is formed. The increase in maximum rate of thrombus generation indicates increased clot growth in diabetic individuals compared to healthy individuals. Lastly, the increase in total thrombus generation shows increased total clot strength. The cumulative effect of these aberrant parameter measures in diabetic individuals is a hypercoagulable state. That is, the increased tendency to develop a clot i.e. larger, denser clots form quicker.

The dysregulated clotting system in diabetic individuals can be attributed to the dysregulated inflammatory *milieu* characteristic of the T2DM diseased state and has previously also been noted as characteristic of the amyloid state found in T2DM [15, 16]. The inflammatory biomarker analyses confirmed a pathological circulating inflammatory profile, where IL-1β, IL-6, IL-8 and sP-selectin were significantly higher in the T2DM group. Platelets will therefore be circulating in a procoagulant and amyloid environment in diabetic individuals. Previously, it was reported that platelets that individuals with T2DM show increased spreading and microparticle formation [40–42]. This agrees with our SEM ultrastructural analysis, which shows activated platelets with significantly increased spreading, and microparticle formation. This was noted in both PRP and WB smears (WB not shown). In addition, confocal microscopy confirmed platelet-derived microparticle formation and shedding of these particles around the actual platelets. Confocal microscopy of T2DM platelets also showed pronounced spreading, activation and aggregation similar to the observation noted in the SEM analyses.

In conclusion, we therefore present evidence that in T2DM there is a comprehensive, systemic and chronic blood hypercoagulability present; and that this is due to the presence of amyloid fibrin(ogen), together with increased circulating inflammatory biomarkers, and a hyperglycaemic state. Platelets undergo structural changes and upregulated receptor expression, along with increased platelet-derived microparticle formation (visible with microscopy techniques). Platelets therefore are excellent, sensitive cellular indicators of the co-occurrence and comorbidity of T2DM and CVD. As microparticles are small in size (less than 200 nm), the size limitation of our flow cytometer excludes them from being measured. We recognize that this as a limitation of our study, and in future studies platelet-derived microparticles can be quantified using nanotracking analysis (Nanosite), which can measure particles as small as 10 nm in diameter [43]. Cumulatively, this provides some mechanistic evidence that pathological states of platelets together with amyloid fibrin(ogen) in T2DM, might underpin the fact that such individuals are at increased risk for cardiovascular events related to increased morbidity and mortality. Furthermore, these results confirm that medical practitioners should not only use the basic pathology tests when diagnosing and treating T2DM individuals, but should also include comprehensive cytokine analyses, thromboelastography as well as platelet function tests. Finally, these novel observations may have good diagnostic potential, particularly if used in a personalized-patient orientated approach, and might even in future have a place in precision medicine, to predict drug/pharmacogenomics/platelet functioning-outcomes.

## Abbreviations

CAF: Central Analytical Facility
CRP: C-reactive protein
CVD: cardiovascular disease
HMDS: hexamethyldisilazane
IDF: International Diabetes Federation
IL: interleukin
K: clot kinetics
MA: maximal amplitude
MRTG: maximum rate of thrombus generation
PBS: phosphate-buffered saline
PPP: platelet-poor plasma
PRP: platelet-rich plasma
R-value: clot reaction time
SEM: scanning electron microscopy
sP-selectin: soluble P-selectin
T2DM: type 2 diabetes mellitus
TEG: thromboelastographic
TMRTG: time to maximum rate of thrombus generation
TNF-a: tumour necrosis factor-alpha
TTG: total thrombus generation
WB: whole blood
